# Impact of Maximizing Css/MIC Ratio on Efficacy of Continuous Infusion Meropenem Against Documented Gram-Negative Infections in Critically Ill Patients and Population Pharmacokinetic/Pharmacodynamic Analysis to Support Treatment Optimization

**DOI:** 10.3389/fphar.2021.781892

**Published:** 2021-12-08

**Authors:** Pier Giorgio Cojutti, Milo Gatti, Matteo Rinaldi, Tommaso Tonetti, Cristiana Laici, Chiara Mega, Antonio Siniscalchi, Maddalena Giannella, Pierluigi Viale, Federico Pea

**Affiliations:** ^1^ SSD Clinical Pharmacology, Department for Integrated Infectious Risk Management, IRCCS Azienda Ospedaliero-Universitaria di Bologna, Bologna, Italy; ^2^ Department of Medical and Surgical Sciences, Alma Mater Studiorum University of Bologna, Bologna, Italy; ^3^ Infectious Diseases Unit, Department for Integrated Infectious Risk Management, IRCCS Azienda Ospedaliero-Universitaria di Bologna, Bologna, Italy; ^4^ Anesthesia and Intensive Care Medicine, IRCCS Azienda Ospedaliero-Universitaria di Bologna, Bologna, Italy; ^5^ Division of Anesthesiology, Department of Anesthesia and Intensive Care, IRCCS Azienda Ospedaliero-Universitaria di Bologna, Bologna, Italy

**Keywords:** css/MIC ratio, efficacy, population pharmacokinetics, pharmacodynamics, continuous infusion meropenem, critically ill patients

## Abstract

**Introduction:** optimal treatment of Gram-negative infections in critically ill patients is challenged by changing pathophysiological conditions, reduced antimicrobial susceptibility and limited therapeutic options. The aim of this study was to assess the impact of maximizing Css/MIC ratio on efficacy of continuous infusion (CI) meropenem in treating documented Gram-negative infections in critically ill patients and to perform a population pharmacokinetic/pharmacodynamic analysis to support treatment optimization.

**Materials and Methods:** Classification and regression tree (CART) analysis was used to identify whether a cutoff of steady-state meropenem concentration (Css)-to-minimum inhibitory concentration (MIC) (Css/MIC) ratio correlated with favorable clinical outcome. A non-parametric approach with Pmetrics was used for pharmacokinetic analysis and covariate evaluation. The probability of target attainment (PTA) of the identified Css/MIC ratio was calculated by means of Monte Carlo simulations. Cumulative fraction of response (CFRs) were calculated against common Enterobacterales, *P. aeruginosa* and *A. baumannii* as well.

**Results:** a total of 74 patients with 183 meropenem Css were included. CART analysis identified a Css/MIC ratio ≥4.63 as cutoff value significantly associated with favorable clinical outcomes. Multivariate regression analysis confirmed the association [OR (95%CI): 20.440 (2.063–202.522); *p* < 0.01]. Creatinine clearance (CL_CR_) was the only covariate associated with meropenem clearance. Monte Carlo simulations showed that, across different classes of renal function, dosages of meropenem ranging between 0.5 and 2 g q6h over 6 h (namely by CI) may grant PTAs of Css/MIC ratios ≥4.63 against susceptible pathogens with an MIC up to the EUCAST clinical breakpoint of 2 mg/L. The CFRs achievable with these dosages were very high (>90%) against Enterobacterales across all the classes of renal function and against *P. aeruginosa* among patients with CL_CR_ < 30 ml/min/1.73 m^2^, and quite lower against *A. baumannii*.

**Discussion:** our findings suggest that Css/MIC ratio ≥4.63 may be considered the pharmacodynamic target useful at maximizing the efficacy of CI meropenem in the treatment of Gram-negative infections in critically ill patients. Dosages ranging between 0.5 g q6h and 2 g q6h by CI may maximize the probability of favorable clinical outcome against meropenem-susceptible Gram-negative pathogens among critically ill patients having different degrees of renal function*.*

## 1 Introduction

Bacterial infections are a major occurrence in critically ill patients, with an overall in-hospital mortality rate of 30% (Vincent et al., 2020). Two-third of these are caused by Gram-negative pathogens (Vincent et al., 2020), and multidrug-resistant (MDR) *Enterobacterales, Pseudomonas aeruginosa,* and *Acinetobacter baumannii* have considerably risen in recent years (MacVane, 2017; Vincent et al., 2020).

The new approved beta-lactam/beta-lactamase inhibitors (BL/BLIs) ceftazidime/avibactam and ceftolozane/tazobactam (Yahav et al., 2020), and the new siderophore cephalosporin cefiderocol (Syed, 2021) have considerably improved the therapeutic armamentarium against carbapenem resistant Gram-negative infections (Bassetti et al., 2018; Rodriguez-Bano et al., 2018; Gatti et al., 2021). However, meropenem still remains a valuable option for the treatment of severe infections due to extended-spectrum beta-lactamases (ESBLs)-producing *Enterobacterales* (Rodriguez-Bano et al., 2018; Gatti et al., 2021; Tamma et al., 2021) and susceptible strains of *Pseudomonas aeruginosa* and *Acinetobacter baumannii* (Bassetti et al., 2018; Rodriguez-Bano et al., 2018; Gatti et al., 2021).

On the one hand, none of the novel BL/BLIs and/or cefiderocol were found to be superior to meropenem in the management of Gram-negative nosocomial pneumonia, complicated-intraabdominal (cIAIs) and/or urinary tract infections (cUTIs) (Solomkin et al., 2015; Mazuski et al., 2016; Torres et al., 2018; Kollef et al., 2019; Wunderink et al., 2021). On the other hand, meropenem showed significantly lower 30-days mortality rate compared to piperacillin-tazobactam in the treatment of ceftriaxone-resistant *Escherichia coli* and/or *Klebsiella pneumoniae* bloodstream infections (BSIs) (Harris et al., 2018).

The minimum pharmacodynamic target of efficacy for meropenem is considered a time of 40% of the dosing interval during which the plasma concentrations exceed the pathogen MIC (40%t_>MIC_) (Ellis et al., 2005). Indeed, recently a more aggressive target up to 100%t>MIC has been advocated for ensuring optimal efficacy with carbapenems among critically ill patients and for preventing resistance development (Wong et al., 2014; Yu et al., 2018) (Sumi et al., 2019; Cojutti et al., 2020), as recommended by a recent position paper on antimicrobial therapeutic drug monitoring in critically ill patients (Abdul-Aziz et al., 2020).

This target may be reached more easily when prolonged or continuous infusion (CI) administration is applied (Vardakas et al., 2018; Guilhaumou et al., 2019; Gatti and Pea, 2021). This approach may optimize target attainment even when in presence of highly fluctuating inter-patient pharmacokinetic variability that may affect the volume of distribution and/or the clearance of meropenem among this population (Blot et al., 2014). Although it has been suggested that during CI administration of meropenem the steady-state concentration (Css) should be maintained above the MIC of the pathogen (Css > MIC) (Abdul-Aziz et al., 2020), it is still to be fully elucidated which magnitude of the Css/MIC ratio may maximize the efficacy of treatment with meropenem among the critically ill patients.

The aim of this study was to assess the impact of maximizing Css/MIC ratio on efficacy of continuous infusion meropenem in treating severe documented Gram-negative infections in critically ill patients and to perform a population pharmacokinetic/pharmacodynamic analysis for predicting dosages of CI meropenem optimal for this purpose.

## 2 Materials and Methods

### 2.1 Study Design

This was a retrospective monocentric study conducted among critically ill patients who were admitted to the ICUs of the IRCCS Azienda Ospedaliero-Universitaria di Bologna, Italy, and who received CI meropenem for empirical or targeted treatment of Gram-negative-related infections in the period between December 2020 and July 2021.

Meropenem was administered alone or in co-treatment with other antimicrobial agents at the discretion of the infectious disease consultant.

At our Institution, meropenem treatment was started with a loading dose (LD) of 2 g over 2 h immediately followed by a maintenance dose (MD) of 1 g q6h over 6 h in patients with creatinine clearance (CL_CR_) ≥ 60 ml/min/1.73 m^2^ or of 0.5 g q6h in those with CL_CR_ < 60 ml/min/1.73 m^2^. After at least 2 days from starting therapy, patients underwent real-time therapeutic drug monitoring (TDM) coupled with clinical pharmacological advice (CPA) for dose adjustments. TDM of meropenem was routinely performed 5-days a week from Monday to Friday, and CPAs were aimed at achieving an optimal pharmacodynamic target of meropenem. This was defined as a steady-state plasma concentration (Css)-to-minimum inhibitory concentration (MIC) ratio of 4–8 whenever targeted treatment with meropenem was feasible (namely in case of known susceptibility of the microbiological isolate). In the other cases (namely empirical treatment) meropenem Css was targeted at 4-8-fold the EUCAST clinical breakpoint of 2 mg/L against Enterobacterales, *Pseudomonas aeruginosa* and *Acinetobacter baumannii*, namely at 8–16 mg/L (Pea et al., 2012). Stability of CI meropenem was granted by reconstitution of the aqueous solution every 6–8 h with infusion over 6–8 h (Franceschi et al., 2014). Drug dosages were adjusted by using linear scaling, with a minimum dose modification of 125–250 mg.

On the day of TDM assessment, 5 ml of peripheral venous blood was drawn and sent immediately to the laboratory for analysis. Meropenem concentrations were analyzed by means of a liquid chromatography-tandem mass spectrometry (LC–MS/MS) commercially available method (Chromsystems Instruments & Chemicals GmbH, Munich, Germany), with a lower limit of detection of 0.3 mg/L.

The following demographic and clinical data were collected from each patient’s medical record: age, gender, weight, height, SOFA score, type and site of infection, bacterial clinical isolate and susceptibility, serum creatinine, meropenem daily dose and eventual co-treatment with other antimicrobial agents. CL_CR_ was estimated by means of the CKD-EPI formula (Levey et al., 2009).

A stepwise procedure for patient inclusion in this study was adopted. Exclusion criteria were absence of critical illness, meropenem administration by extended or intermittent infusion, blood sampling inconsistency, and application of renal replacement therapy.

### 2.2 Assessment of Clinical Outcome

Clinical outcomes were defined as cured, unchanged or failed according to the treatment response assessed at the end of therapy by the attending physician. A patient was classified as cured if fever disappeared for >48 h, inflammatory biomarkers (C-reactive protein and/or pro-calcitonin) had a consistent decrease from baseline values and/or microbiological eradication was documented. Clinical outcome was defined as unchanged or failed in case of lack of clinical response or of worsening of clinical conditions at the end of therapy, respectively.

### 2.3 CART Analysis of Css/MIC Ratio to Predict Clinical Cure

Classification and regression tree (CART) analysis was used to develop a prediction model useful at identifying the cutoff value of Css/MIC ratio that best correlated with favorable clinical cure in patients with documented infection. In case of patients with multiple isolates, the highest microbiological MIC value was used. Logistic regression analysis was used to explore the correlation existing between drug exposure and/or clinical factors classified as binary variables with the probability of clinical cure. For patients treated with antimicrobial combination therapy, a dichotomous categorical variable was created. Covariates with a *p*-value of <0.20 at univariate analysis were deemed of potential clinical relevance and were included in the multivariate model on the basis of a forward/backward stepwise approach.

### 2.4 Population Pharmacokinetic Modelling

Population pharmacokinetic analysis was conducted by using the non-parametric adaptive grid (NPAG) approach and the algebraic model solver included in the Pmetrics package (version 1.5.0; Laboratory of Applied Pharmacokinetics and Bioinformatics, Los Angeles, CA, USA) of R (version 3.4.4) (Neely et al., 2012). A one-compartment base model with zero-order administration and first-order elimination from the central compartment was developed. Pharmacokinetic models with more than one compartment were not tested as time-concentration data come only from patients treated with CI meropenem. Maximum a posteriori (MAP)-Bayesian estimates of meropenem clearance (CL) and volume of distribution (V) were determined in each patient.

The possible association of parameter-covariate was explored according to a forward-backward procedure by testing by means of linear regression some biologically plausible potential clinical covariates, namely age, height, weight, gender, CL_CR_, with the median posterior estimates of meropenem pharmacokinetic parameters.

Comparisons of the performances of the models were evaluated by calculating the objective function value (OFV), as well as the Akaike information criteria (AIC) and the Bayesian information criteria (BIC). A decrease of at least 3.84 points in the OFV coupled with a decrease of the AIC and the BIC values were considered for including the covariate into the basic model. The goodness of fit of the observed vs. predicted plot and the coefficient of determination of the linear regression of the observed vs. predicted concentration were also considered. Internal model validation was performed by means of a visual predictive check (VPC) and by calculating the normalized prediction distribution errors (NPDE). The VPC plot was based on 1,000 simulations per subject in the original population, and by overlaying the observed plasma concentrations with the 95% CIs of the simulated 5th, 25th, 50th, 75th, and 95th percentiles. The 95% CI of each parameter in the final model were simulated from 1,000 non-parametric bootstrap with replacement from the weighted marginal distribution of each parameter.

Assay error in the population model was estimated by means of the laboratory inter-day variability assay data. A first-order polynomial equation was estimated by linear regression of the means and associated standard deviations (SD) at five known meropenem concentrations. The coefficients of the four-term polynomial functions were 0.0798, 0.0927,0 and 0. Extra-process noise was captured with a gamma model (γ = 2).

### 2.5 Monte Carlo Simulation Analysis

One-thousand subjects Monte Carlo simulations were conducted by using Pmetrics to estimate the meropenem Css at 72 h achievable with eight dosing regimens of CI meropenem (0.25 g q6h CI, 0.5 g q6h CI, 1 g q8h CI, 1 g q6h CI, 1.25 g q6h CI and 1.5 g q6h CI, 2 g q6h CI and 2.5 g q6h CI). Variability of the significant covariates included in the final population model was considered by calculating the correlation matrix between all covariates and Bayesian posterior estimates values.

The probabilities of target attainment (PTAs) of the identified cutoff value of Css/MIC ratio with the various meropenem doses were calculated. The cumulative fractions of response (CFRs) achievable against the EUCAST MIC distribution of *Escherichia coli*, *Klebsiella pneumoniae*, *Enterobacter cloacae*, *Pseudomonas aeruginosa* and *Acinetobacter baumannii* were calculated as well The European Committee on Antimicrobial Susceptibility Testing, 2021. PTAs and CFRs % were defined as optimal when ≥90% (Turnidge and Paterson, 2007).

### 2.6 Statistical Analysis

The Kolmogorov-Smirnov test was used to assess whether data were normally or non-normally distributed. Accordingly, the mean plus SD or median with IQR was used in the descriptive statistics. A *p* value of <0.05 was required to achieve statistical significance. All statistical analyses were performed using R version 3.4.4 (R Foundation for Statistical Computing, Vienna, Austria).

## 3 Results

### 3.1 Patient Characteristics

**FIGURE 1:**
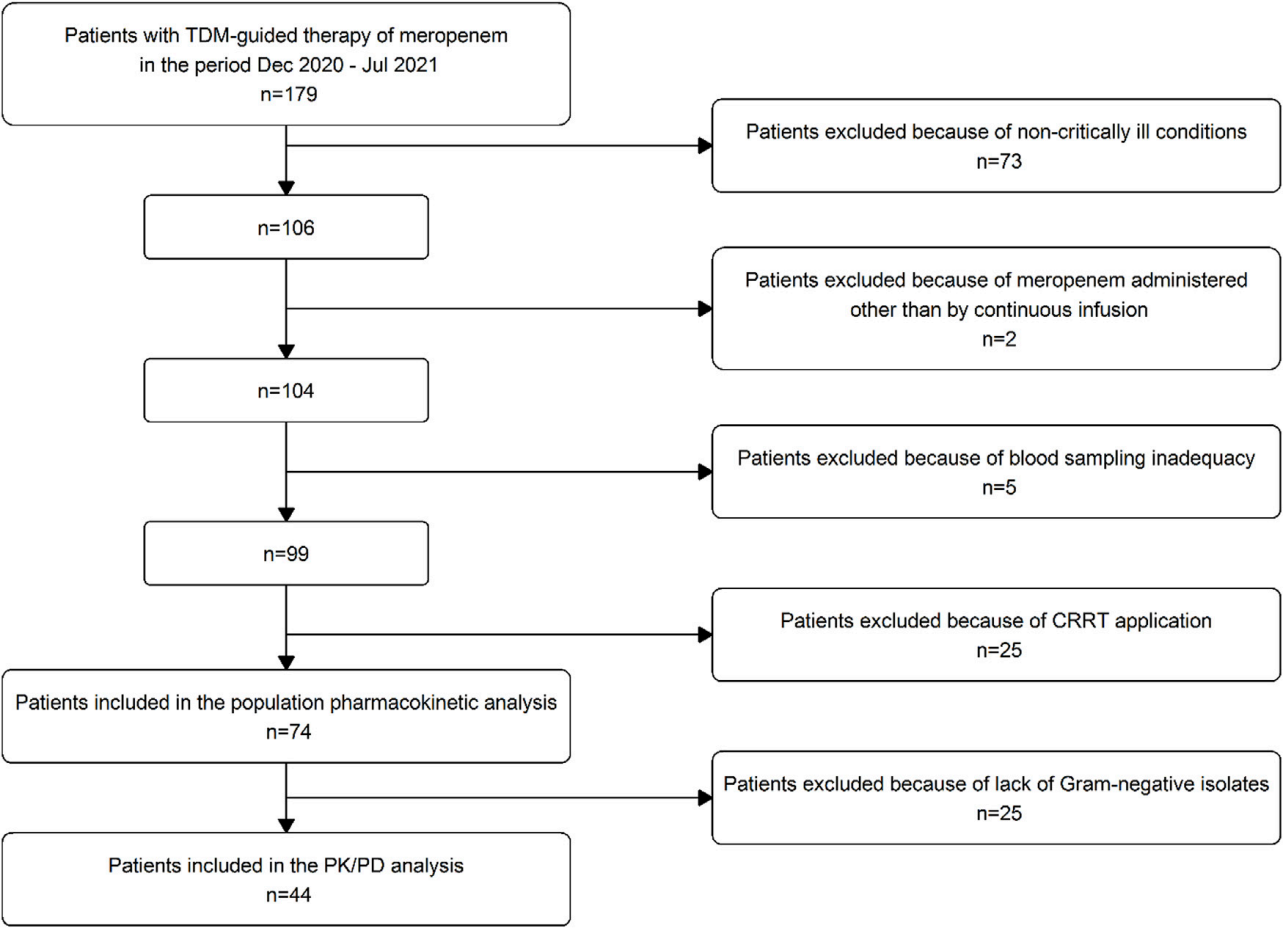
Flow chart of patient inclusion and exclusion criteria.

A total of 179 patients were screened and 74 were included in the study (Figure 1). Patient’s demographic and clinical characteristics are reported in Table 1. Median (IQR) age, weight and CL_CR_ were 60.1 (12–86) years, 79 (50–160) kg and 91.5 (7–192) ml/min/1.73 m^2^, respectively. Ten out of 74 patients (13.5%) had augmented renal clearance (defined as CL_CR_ ≥ 130 ml/min/1.73 m^2^) at start of treatment. Overall, hospital-acquired pneumonia and bloodstream infections were the most prevalent infections (56/74, 75.7%).

**TABLE 1:** Population characteristics.

Patient demographic	
Age (yrs [mean ± SD])	60.1 ± 15.0
Gender (male/female) [n (%)]	52/22 (70.3/29.7)
Body weight (kg) [median (IQR)]	79.0 (68.5–89.5)
Body mass index (kg/m^2^) [median (IQR)]	26.0 (23.6–29.4)
CL_CR_ (ml/min/1.73 m^2^)^a^[median (IQR)]	91.5 (51.2–114.9)
Augmented renal clearance [n (%)]	10 (13.5)
SOFA score^a^ [median (IQR)]	7.0 (5.0–10.0)
Indication for meropenem use [n (%)]	
HAP/VAP	37 (50.0)
BSI	19 (25.7)
cIAI	11 (14.9)
cUTI	4 (5.4)
Meningitis	2 (2.7)
Bone and joint infection	1 (1.4)
Patients with identified microbiological isolates [n (%)]	44 (59.5%)
Meropenem treatment	
Median dose (g/daily)	1 q8h (0.5 g q6h—1 g q6h)
Length of therapy [days; median (IQR)]	10.5 (7–15)
No. of TDM assessments per patient	2 (1–3)
Meropenem Css (mg/L)	14.1 (9.0–21.3)
Combination therapy [n (%)]	11 (14.9%)
Clinical outcome [n (%)]	
Cured	42 (56.8%)
Failed	26 (35.1%)
Dead for other reasons	6 (8.1%)

a At baseline.

Data are presented as median (IQR) for continuous variables and as n (%) for dichotomous variables.

Median (IQR) total daily dose of meropenem was 1 g q8h CI (0.5 g q6h—1 g q6h), with a median (IQR) duration of treatment of 10.5 (7–15) days. Twenty-five out of 74 patients (33.8%) underwent dose adjustments. Among these, the dose was decreased in 14 cases (56.0%), increased in 8 cases (32.0%), and both increased and decreased during treatment in other 3 patients (12.0%). Overall, clinical cure was obtained in 42/74 (56.8%) of cases.

### 3.2 CART Analysis of Css/MIC Ratio to Predict Clinical Cure

Forty-four patients had documented Gram-negative bacterial infections and were deemed eligible for the CART analysis. A total of 77 bacterial strains were yielded. *P. aeruginosa*, *K. pneumoniae*, *E. coli* and *A. baumannii* accounted for most of them (53/77; 68.8%) (Table 2). Broncho alveolar lavage and blood were the most frequent primary sources of infection (75.3%, 58/77). Most of the patients with documented Gram-negative bacterial infections (84.1%, 37/44) had meropenem in monotherapy, and 61.4% of them (27/44) were cured.

**TABLE 2:** Gram-negative pathogens (*n* = 77 from 44 patients) included in the pharmacokinetic/pharmacodynamic analysis.

Pathogen	No. of isolates	MIC range (mg/L)
Enterobacterales		
*Klebsiella pneumoniae*	15	0.125–1
*Escherichia coli*	12	0.125–1
*Enterobacter aerogenes*	5	0.125–0.25
*Enterobacter cloacae*	5	0.125–1
*Proteus mirabilis*	4	0.125
*Citrobacter freundii*	2	0.125
*Klebsiella oxytoca*	2	0.125
*Enterobacter absuriae*	1	0.125
*Enterobacter kobei*	1	0.125
*Klebsiella variicola*	1	0.125
*Morganella morgannii*	1	0.125
*Serratia marcescens*	1	0.125
Non-fermenting Gram-negatives		
*Pseudomonas aeruginosa*	17	0.125–32
*Acinetobacter baumannii*	9	0.125- ≥32
*Burkholderia cepacia*	1	0.5

**FIGURE 2:**
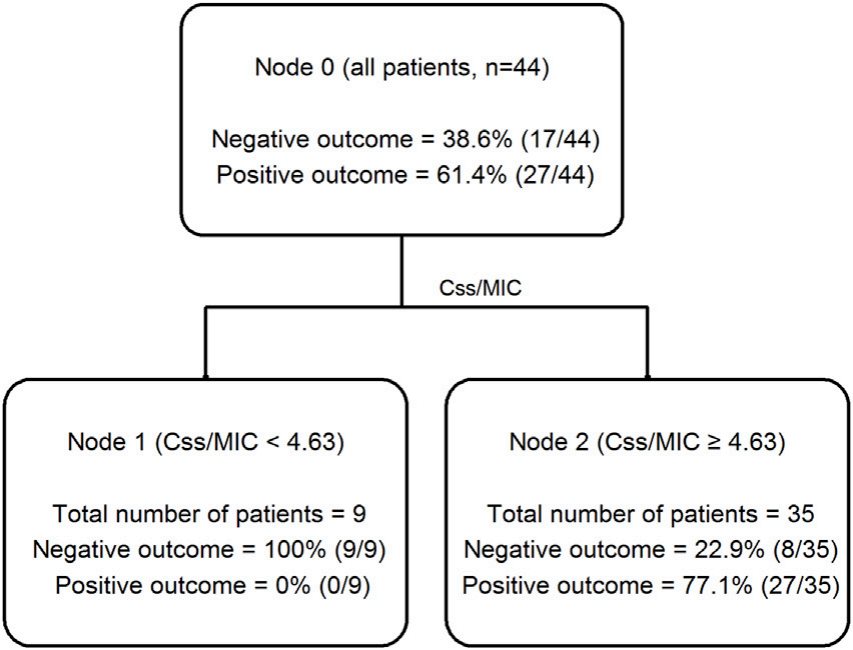
Classification and regression tree (CART) analysis of the partition of clinical outcome depending on meropenem Css/MIC ratio in critically ill patients (*n* = 44).

CART analysis identified a cutoff value of Css/MIC ratio ≥4.63 as valuable predictor of favorable clinical cure (Figure 2). Clinical cure was observed in 26 out of 35 patients (74.3%) who had Css/MIC ratio equal or above this threshold, and only in 1 out of the 9 (11.1%) having Css/MIC ratio below it.

Table 3 summarizes the logistic regression analysis of potential covariates associated with clinical cure. Among the different variables tested at multivariate regression analysis, only meropenem Css/MIC ratio ≥4.63 was significantly associated with clinical cure (OR 20.440; 95%CI 2.063–2,102.522; *p* = 0.010), with an area under the ROC curve of 0.78.

**TABLE 3:** Univariate and multivariate logistic regression analysis of variables associated with favorable clinical outcome (*n* = 44).

	Univariate analysis	P	Multivariate analysis	P
Variable	OR (95% CI)		OR (95% CI)	
Age >65 years	0.523 (0.153–1.792)	0.302		
Gender (male vs. female)	0.998 (0.262–3.744)	0.988		
BMI^a^ >30 kg/m^2^	1.061 (0.218–5.151)	0.942		
ARC^b^	2.234 (0.574–8.691)	0.246		
Meropenem Css/MIC ratio ≥4.63	29.250 (3.200–267.366)	0.003	**20.440 (2.063–202.522)**	**0.010**
SOFA score >10	0.344 (0.053–2.215)	0.200	0.299 (0.036–2.471)	0.262
Combination therapy	0.001 (-0.001–0.001)	1.000		

a BMI, body mass index.

b ARC, augmented renal clearance (defined as estimated creatinine clearance ≥130 ml/min/1.73 m^2^).

### 3.3 Population Pharmacokinetics Analysis

A total of 183 meropenem Css were included in the population PK model. The one-compartment base model provided a good fit of data (R^2^ of observed vs. predicted concentrations = 0.796) with OFV, AIC and BIC of 1196, 1202 and 1212, respectively.

The two covariates that significantly improved the model performances were CL_CR_ on meropenem CL and patient weight on meropenem V. After inclusion of these covariates into the basic model, the R^2^ of the regression value of the observed versus individual predicted concentrations increased to 0.817, and the values of the OFV, AIC and BIC decreased to 1132, 1142 and 1158, respectively.

**FIGURE 3:**
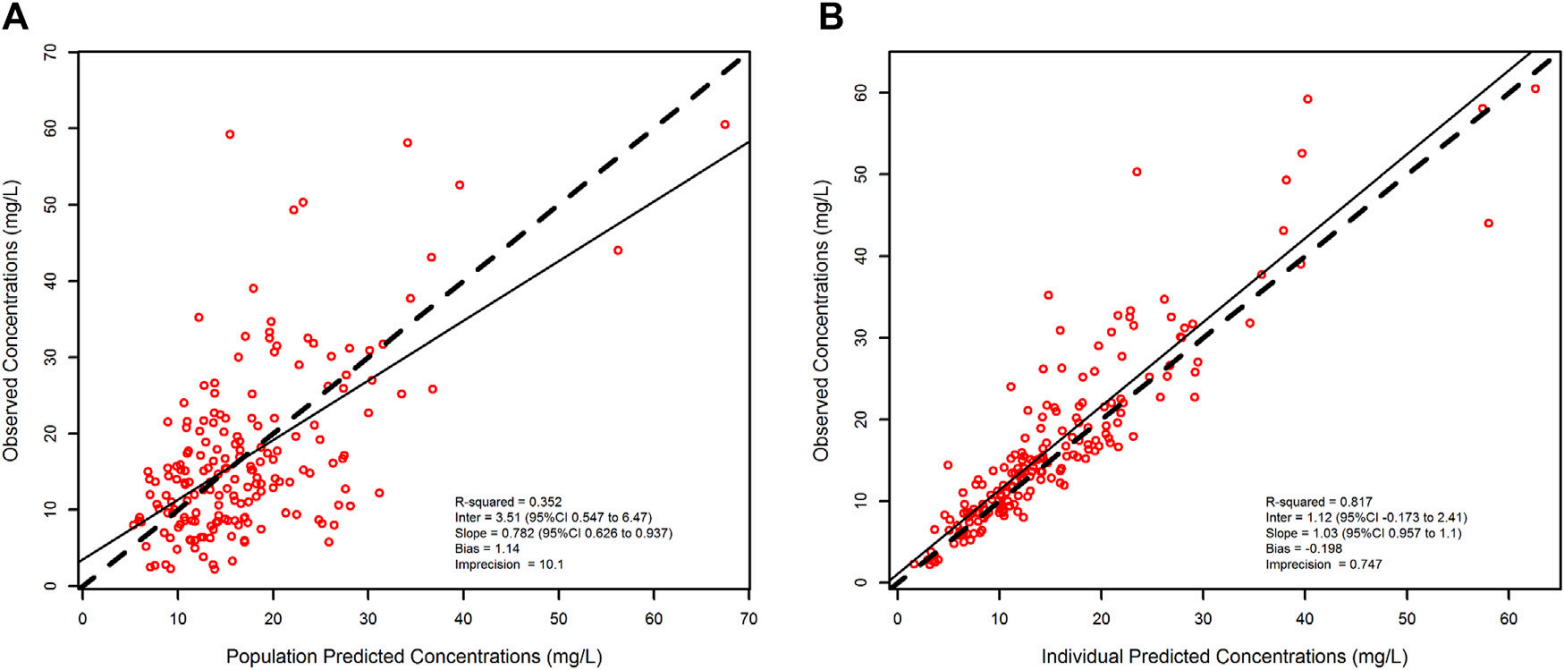
Scatter and linear fit plot for the final population pharmacokinetic model. Observed versus population-predicted plasma concentrations (left panel) and observed versus individual-predicted plasma concentrations (right panel).

Figure 3 shows the relationship between meropenem observed vs. predicted concentration at a population level (R^2^ = 0.352, bias = 1.14, imprecision = 10.1) and after Bayesian optimization (R^2^ = 0.817, bias = −0.198, imprecision = 0.747).

The final model was parameterized as follows:

$$CLi=\theta_{1}+\theta_{2}\times CL_{CRi}$$

$$Vi=\theta_{3}*{\left(BWi\right)}^{\wedge \theta_{4}}$$

where CLi and Vi are meropenem clearance and volume of distribution, respectively, of the ith subject, θ_1_ is the clearance (intercept) when CL_CR_ = 0, θ_2_ is the slope estimate reflecting the change in clearance per unit change in CL_CR_, θ_3_ is the distribution volume when BW = 1, θ_4_ is the positive exponent estimate reflecting the change in the natural log of volume per unit change in the natural log of BW. CL_CRi_ is the creatinine clearance of the ith subject. BWi is the body weight of the ith subject. The parameter estimates of the final model are summarized in Table 4.

**TABLE 4:** Parameter estimates of continuous infusion meropenem for the final population pharmacokinetic model.

	Final model		Bootstrap			
	Mean parameter estimate	CV (%)	Median parameter estimate	95% CI parameter estimate	Median CV (%)	95% CI CV (%)
$\text{CL }\left(\text{L}/\text{h}\right)={\text{θ}}_{1}+{\text{θ}}_{2}\times$ CL_CR_						
θ_1_	1.040	77.016	0.908	0.186–1.860	114.206	22.809–148.062
θ_2_	0.103	66.074	0.080	0.058–0.137	72.102	20.935–111.727-
$\text{V }\left(\text{L}\right)={\text{θ}}_{3}\times {\text{BW}}^{{\text{θ}}_{4}}$						
θ_3_	7.343	46.824	9.288	6.091–9.364	11.339	1.212–51.945
θ_4_	0.612	59.146	0.554	0.500–0.611	34.904	14.258–64.085

BW, total body weight; CL, meropenem clearance; CL_CR_, creatinine clearance estimated by means of the CKD-EPI formula; CI, confidence interval; CV, coefficient of variation; V, meropenem volume of distribution.

θ_1_ and θ_2_ are the intercept and slope estimates, respectively, of the linear regression between meropenem CL, and CL_CR_., θ_3_ is the distribution volume coefficient when total body weight = 1 and θ_4_ is the positive exponent estimate of the power relationship between V and body weight.

**FIGURE 4:**
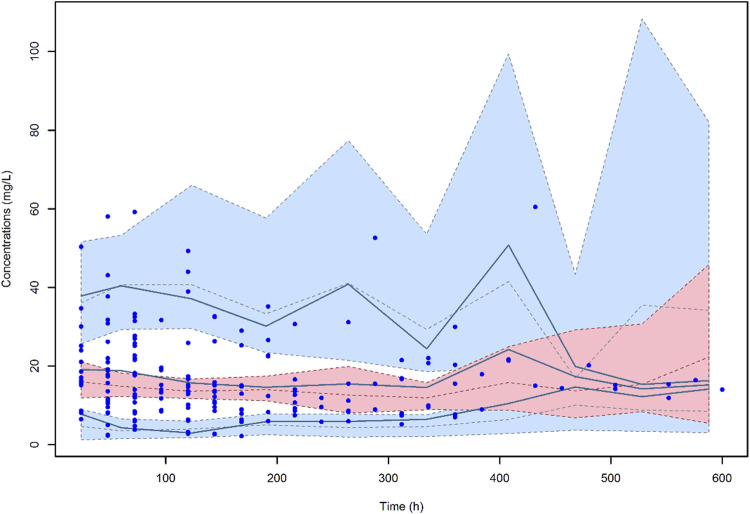
Visual predictive check (VPC) of meropenem plasma concentration-versus-time for the final model. The continuous lines indicate the 10th, 50th and 90th percentiles for observed data, while the shaded areas represent 90% prediction intervals from the corresponding percentiles calculated from simulated data.

The VPC of the final model (Figure 4) showed that the distribution of the observed concentrations was consistent with that of the predicted concentrations, as the 95.6% of the observations were within the 95% CI of model predictions. The normal distribution of NPDE (*p* = 0.655 at the Shapiro-Wilks normality test) confirmed the adequacy of the model. Median (IQR) total CL and V of meropenem were 7.27 (4.53–10.41) L/h and 20.0 (17.16–23.59) L, respectively.

### 3.4 Monte Carlo Simulation Analysis

**FIGURE 5:**
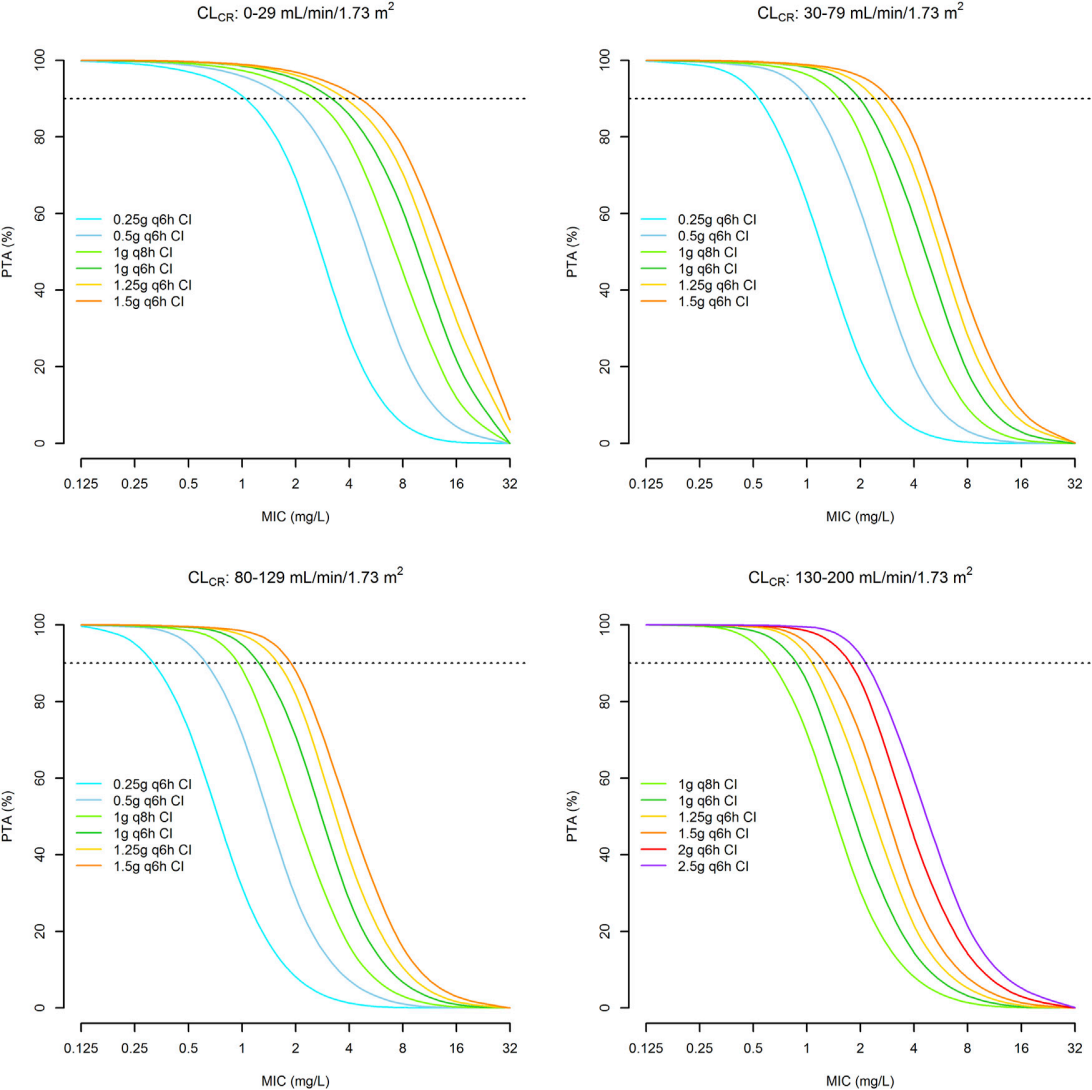
Probability of achieving a Css/MIC value of ≥4.63 with continuous infusion dosages of meropenem in relation to different classes of renal function and the MIC values. Horizontal dotted line identifies the threshold for optimal PTA (≥90%).

A total of thirty-two 1,000-subject Monte Carlo simulations were conducted with the tested doses of CI meropenem across four different classes of CL_CR_ (0–29, 30–79, 80–130, 130–200 ml/min/1.73 m^2^). CL_CR_ followed a uniform distribution within each class. Figure 5 shows the PTAs of a Css/MIC ratio ≥4.63 against *E. coli*, *K. pneumoniae*, *E. cloacae*, *P. aeruginosa* and *A. baumannii*. Optimal PTAs at the EUCAST clinical breakpoints of 2 mg/L were obtained with meropenem dosages of 0.5 g q6h CI, 1 g q6h CI, 1.5 g q6h CI and 2 g q6h CI in patients with CL_CR_ of 0–29, 30–79, 80–129 and 130–200 ml/min/1.73 m^2^, respectively.

Table 5 summarizes the CFRs at Css/MIC ratio of ≥4.63 achievable with incremental dosages of CI meropenem in different classes of renal function against the EUCAST MIC distributions of *E. coli*, *K. pneumoniae*, *E. cloacae*, *P. aeruginosa* and *A. baumannii*. Optimal CFRs were granted against *E. coli*, *K. pneumoniae* and *E. cloacae* just with a dosing regimen of 0.25 g q6h CI across all of the classes of renal function. Against *P. aeruginosa* CFRs were optimal only in patients with CL_CR_ of 0–29 ml/min/1.73 m^2^ with a meropenem dosage as high as 1.25 g q6h CI, whereas in all of the other classes of renal function were suboptimal, ranging between 82.71% and 86.84. Similarly, CFRs against *A. baumannii* were always suboptimal, and ranged from 77.02 to 87.62% even when considering the highest dosages in all of the classes of CL_CR_.

**TABLE 5:** Cumulative fraction of response (CFR) achievable with incremental dosages of continuous infusion (CI) meropenem at different classes of renal function for a pharmacodynamic target of plasma steady-state (Css)/minimum inhibitory concentration (MIC) ratio of 4.63 against the EUCAST MIC distribution of *Escherichia coli*, *Klebsiella pneumoniae*, *Enterobacter cloacae*, *Pseudomonas aeruginosa* and *Acinetobacter baumannii*.

CI meropenem dosages at classes of renal function	*E. coli*	*K. pneumoniae*	*E. cloacae*	*P. aeruginosa*	*A. baumannii*
CL_CR_: 0–29 ml/min/1.73 m^2^					
0.25 g q6h CI	99.05	99.09	99.05	77.01	70.82
0.5 g q6h CI	99.40	99.36	99.40	83.36	77.03
1 g q8h CI	99.55	99.47	99.55	86.81	80.29
1 g q6h CI	99.65	99.54	99.65	89.35	83.11
1.25 g q6h CI	99.72	99.59	99.72	91.11	85.33
1.5 g q6h CI	99.77	99.64	99.77	92.62	87.42
CL_CR_: 30–79 ml/min/1.73 m^2^					
0.25 g q6h CI	99.76	98.66	98.41	65.86	56.75
0.5 g q6h CI	99.83	99.11	99.11	76.11	69.51
1 g q8h CI	99.86	99.27	99.32	80.31	74.51
1 g q6h CI	99.87	99.37	99.45	83.42	77.47
1.25 g q6h CI	99.88	99.43	99.53	85.38	79.22
1.5 g q6h CI	99.89	99.48	99.58	86.84	80.51
CL_CR_: 80–129 ml/min/1.73 m^2^					
0.25 g q6h CI	99.68	98.23	97.53	54.73	45.02
0.5 g q6h CI	99.78	98.85	98.71	68.88	60.16
1 g q8h CI	99.82	99.06	99.60	74.84	67.59
1 g q6h CI	99.85	99.21	99.26	78.63	72.49
1.25 g q6h CI	99.86	99.29	99.36	80.93	75.21
1.5 g q6h CI	99.87	99.35	99.43	82.71	77.02
CL_CR_: 130–200 ml/min/1.73 m^2^					
1 g q8h CI	99.79	98.86	98.75	69.26	60.59
1 g q6h CI	99.82	99.03	99.01	73.74	66.02
1.25 g q6h CI	99.84	99.14	99.18	76.84	70.06
1.5 g q6h CI	99.85	99.22	99.28	78.99	72.83
2 g q6h CI	99.87	99.33	99.41	82.08	76.43
2.5 g q6h CI	99.88	99.40	99.50	84.34	78.65

CL_CR_, creatinine clearance.

## 4 Discussion

In this study we identified that a Css/MIC ratio ≥4.63 predicted efficacy of CI meropenem against documented Gram-negative infections in critically ill patients and we carried out a population pharmacokinetic/pharmacodynamic analysis to support treatment optimization.

To the best of our knowledge, this is the first study that identified at CART analysis a threshold of Css/MIC ratio as valuable predictor of efficacy of CI meropenem against documented Gram-negative infections in critically ill patients. Interestingly, this value is quite similar to those of C_min_/MIC ratio found to predict efficacy with intermittent infusion (II) meropenem either among 46 critically ill patients with Gram-negative bloodstream infections (>4.95) (Wong et al., 2020) or among 101 patients with lower respiratory tract infections (>5) (Li et al., 2007). Additionally, it’s worth noting that a C_min_/MIC ratio of similar extent (>3.8) was also significantly associated with regrowth prevention and avoidance of resistance development in an *in vitro* hollow fiber infection model that tested the development of resistance against *K. pneumoniae* and *P. aeruginosa* strains exposed to intermittent dosing regimens of ceftazidime, cefepime and meropenem (Tam et al., 2017).

We found that the magnitude of the pharmacodynamic target needed for maximizing the efficacy of meropenem during CI use (Css/MIC ratio) was very similar to the one that was shown to be needed during II use (C_min_/MIC ratio). This has some relevant clinical implications. First, it allows to speculate that when meropenem is administered by CI the daily doses needed to achieve the desired pharmacodynamic target of efficacy should be lower compared to those needed for II. Second, when considering the same daily dose of meropenem, CI administration may ensure Css/MIC ratios against a given pathogen that are higher than the C_min_/MIC ratios that could be achieved with II (Vardakas et al., 2018; Guilhaumou et al., 2019). Additionally, CI administration may avoid unnecessary fluctuations of concentrations and prevent too high peak levels that could be potentially associated with toxicity. Overall, in several studies extended and/or CI administration were shown to be superior compared to intermittent infusion in attaining a given pharmacodynamic target of efficacy and in improving clinical outcomes with beta-lactams among the critically ill patients (Lorente et al., 2006; Chytra et al., 2012; Wong et al., 2014; Roberts et al., 2016; Yu et al., 2018).

Population pharmacokinetic analysis found that the only covariate significantly associated to meropenem CL was CL_CR_. This is in agreement with previous findings and it is consistent with meropenem being eliminated mainly by the renal route. Different studies assessed the population pharmacokinetics of CI meropenem among critically ill patients. Meropenem CL estimate in our study (7.27 L/h) is closer to the values reported among two of them (Minichmayr et al., 2018) (Thalhammer et al., 1999). In a retrospective study carried out among195 critically ill patients with an estimated CL_CR_ of 65 ml/min, meropenem CL estimate was 7.71 L/h (Minichmayr et al., 2018). In a crossover prospective study carried out among 15 critically ill patients mainly affected by pneumonia and meropenem CL was 7.7 L/h (Thalhammer et al., 1999). Other studies found very variable meropenem CL values in relation to different CL_CR_ estimates. Kees et al. observed meropenem CL of 10.8 L among 32 surgical ICU patients with a measured CL_CR_ of 65.3 ml/min (Kees et al., 2016). Similar CL values (were obtained among 123 critically ill patients with estimated CL_CR_ of 93.9–106.9 ml/min 10.17–11.19 L/h) (Pea et al., 2012), and among 21 ICU patients with a measured CL_CR_ of 74.9 ml/min (9.89 L/h) (Dhaese et al., 2019). Finally, a recent retrospective study reported a population CL estimate of 4.8 L/h among 58 critically ill patients, 26 of whom were undergoing continuous renal replacement therapy (O'Jeanson et al., 2021). As far as the estimate of Vd is concerned, our Vd estimate (20.0 L) is consistent with that reported by Thalhammer (25.9 L) (Thalhammer et al., 1999), and lower of that reported by O’Jeanson (43 L) (O'Jeanson et al., 2021).

Monte Carlo simulation showed that meropenem dosages ranging from 0.5 g q6h to 2 g q6h by CI may support treatment optimization of CI meropenem against Gram-negative infections among critically ill patients with various degrees of renal function. These dosages, by always allowing optimal PTAs of Css/MIC ≥4.63 against strains with an MIC up to the EUCAST clinical breakpoint of 2 mg/L, may predict efficacy against all of the susceptible *Enterobacterales*, *P. aeruginosa* and *A. baumannii*. These dosages resulted in optimal CFRs against the EUCAST MIC distribution of the most common Enterobacterales across all of the classes of renal function as well. Conversely, only suboptimal CFRs were obtained against the MIC distributions of *P. aeruginosa* and *A. baumannii* across all of the classes of renal function, except than in the case of *P. aeruginosa* in patients with renal failure. However, it should not be overlooked that, according the EUCAST MIC distributions of *P. aeruginosa* and *A. baumannii*, almost three-quarter of the strains are still susceptible to meropenem (79.4 and 74.8%, respectively). Consequently, meropenem may still represent a valuable therapeutic option in the majority of *P. aeruginosa* and *A. baumannii* related infections. Conversely, in the remaining cases, ceftolozane/tazobactam or cefiderocol could represent valuable alternatives.

We recognize that this study has some limitations. The retrospective design, the limited number of TDM assessments per patient and the fact that CL_CR_ was estimated rather than measured must be acknowledged. These could have concurred in some unexplained variability of the pharmacokinetic parameters. Conversely, the finding of a Css/MIC ratio helpful in predicting efficacy of CI meropenem against documented Gram-negative infections in critically ill patients and the population pharmacokinetic/pharmacodynamic analysis to support treatment optimization are valuable points of strength.

In conclusion, our findings showed that a Css/MIC ≥4.63 is associated with clinical cure among critically ill patients treated with CI meropenem for documented Gram-negative infections. Meropenem dosages ranging between 0.5 and 2 g q6h by CI may support treatment optimization in different classes of renal function, but real–time TDM coupled with clinical pharmacological advices may still represent an invaluable tool for tailoring optimal treatment in each single patient.

## Data Availability

The raw data supporting the conclusion of this article will be made available by the authors, without undue reservation.
